# Anaerobic metabolism associated with traumatic hemorrhagic shock monitored by microdialysis of muscle tissue is dependent on the levels of hemoglobin and central venous oxygen saturation: a prospective, observational study

**DOI:** 10.1186/1757-7241-22-11

**Published:** 2014-02-05

**Authors:** Filip Burša, Leopold Pleva

**Affiliations:** 1 Department of anesthesiology and intensive care medicine, University Hospital Ostrava, Faculty of Medicine Universitas Ostrava, 17 listopadu, 1790 Ostrava-Poruba, Czech Republic; 2 Traumatology Centre, University Hospital Ostrava, Faculty of Medicine Universitas Ostrava, 17 listopadu, 1790 Ostrava-Poruba, Czech Republic

**Keywords:** Microdialysis, Shock, Lactate, Hemoglobin

## Abstract

**Background:**

Traumatic hemorrhagic shock resulting in tissue hypoxia is a significant cause of morbidity and mortality in polytraumatized patients. Early identification of tissue hypoxia is possible with microdialysis. The aim of this study was to determine the correlation between a marker of tissue hypoxia (L/P; lactate to pyruvate ratio) and selected parameters of systemic oxygen delivery (Hb; hemoglobin) and oxygen extraction (ScvO_2_; central venous oxygen saturation). We also investigated the severity of tissue hypoxia over the course of care.

**Methods:**

Adult patients with traumatic hemorrhagic shock were enrolled in this prospective, observational study. Microdialysis of the peripheral muscle tissue was performed. Demographic data and timeline of care were collected. Tissue lactate, pyruvate, glycerol, glucose levels, hemoglobin, serum lactate and oxygen saturation of the central venous blood (ScvO_2_) levels were also measured.

**Results:**

The L/P ratio trend may react to changes in systemic hemoglobin levels with a delay of 7 to 10 hours, particularly when systemic hemoglobin levels are increased by transfusion. Decrease in tissue L/P ratio may react to increase in ScvO_2_ with a delay of up to 10 hours, and such a decrease may signify elimination of tissue hypoxia after transfusion. We also observed changes in the L/P trend in the 13 hours preceding a change in the hemoglobin level. Fluid administration, which is routinely used as a first-line treatment of hypovolemic shock, can cause hemodilution and decreased hemoglobin. When ScvO_2_ decreases, increase in L/P ratio may precede the ScvO_2_ trend by 10 or 11 hours. An increase in the L/P ratio is an early warning sign of insufficient tissue oxygenation and should lead to intensive observation of hemoglobin levels, ScvO_2_ and other hemodynamic parameters. Patients who were treated more rapidly had lower maximal L/P values and a lower degree of tissue ischemia.

**Conclusion:**

The L/P ratio is useful to identify tissue ischemia and can estimate the effectiveness of fluid resuscitation. An increase in the L/P ratio is an early warning sign of inadequate tissue oxygenation and should lead to more detailed hemodynamic and laboratory monitoring. This information cannot usually be obtained from global markers.

## Background

Polytrauma is the most frequent cause of death in adults up to 40 years old, and the incidence of death by trauma is 60-80/100,000 traumas in developed countries. A key cause of morbidity and mortality in polytraumatized patients is hemorrhagic shock. Hemorrhage leads to decreased oxygen delivery to tissues and causes severe tissue hypoxia and oxygen debt, which is a core cause of multi-organ failure [1]. Hemoglobin is one of the determinants of oxygen delivery, and one of the first compensatory reactions to a decrease in hemoglobin is an increase in oxygen extraction and thus decreased central venous oxygen saturation (ScvO_2_). The aim of this study was to investigate the dependence of tissue hypoxia on hemoglobin and ScvO_2_ levels.

Adequate therapy of hemorrhagic shock is guided by the clinical status of the patient and global parameters of circulation and metabolism. Identification of both occult and inadequately resuscitated shock is a major clinical problem, and occult shock can be present with normal hemodynamics [2]. Markers for assessing the degree and duration of shock are still controversial. Clinicians can use heart rate, blood pressure, urine output, invasive hemodynamic monitoring of cardiac output including oxygen delivery and consumption, central venous oxygen saturation or metabolic markers such as lactate and base deficit to guide their therapy. Elevated blood lactate levels are associated with increased mortality and morbidity and can be elevated without clinical signs of shock [3]. Shock is a state of hypoperfusion at the cellular level that occurs when the delivery of oxygen (DO_2_) to tissues falls below the tissue oxygen consumption (VO_2_). Oxygen delivery is dependent on blood flow and arterial oxygen content. An imbalance between the delivery and consumption of oxygen leads to the development and accumulation of an oxygen debt. Different DO_2_ distributions to various tissue beds may result in isolated organ ischemia before the occurrence of whole-body ischemia and before the detection of elevated systemic markers of ischemia. Decreased DO_2_ to critical oxygen delivery levels, when increased oxygen extraction past the extraction limit is not possible, causes a proportional decrease in VO_2_ and the emergence of an oxygen debt. This phenomenon leads to multi-organ dysfunction syndrome, which is a leading cause of morbidity and mortality in trauma. Resuscitation efforts should be focused on preventing further oxygen debt accumulation and repayment of the current oxygen debt. The main problem is the resolution of the emerging oxygen debt because its origin is at the cellular level [4]. Changes at the level of the macrocirculation are preceded by changes at the level of individual cells, the smallest capillaries and the extracellular fluid. Neither the effective supply of oxygen required by cells nor their ability to utilize that oxygen are known. Timely recognition of changes in metabolism at the cellular level may lead to more effective treatment of patients.

Resuscitation management in polytraumatized patients with hemorrhagic shock has been well described [5]. The major tasks are rapidly and correctly identifying severely traumatized patients and the early identification of patients with severe hemorrhagic shock. In addition to vital signs, either serum lactate levels or base deficit measurements are recommended as sensitive tests to estimate the extent of the shock. It is expected that lactate levels will track the oxygen debt, but in some clinical conditions, lactate levels may normalize without tissue oxygen debt resolution [6]. The key issue is the type and amount of fluid therapy that is needed to restore oxygen delivery and repay the oxygen debt. Low-volume resuscitation is recommended, including the use of vasopressors and fluid administration, with a hemoglobin target of 7 to 9 g/dl [5]. The administration of fluids in hemorrhagic shock can be sufficient to maintain adequate oxygen delivery to tissues but may not be high enough to dislodge clots, leading to deterioration of the coagulation pathway and increased blood loss. Eliminate of tissue hypoxia based on L/P normalization could be an exact end-point for fluid administration. Improvements in monitoring techniques could influence outcomes in these challenging patients [7].

Capillaries and the extracellular fluid envelope cells and form a network through which nutrients and oxygen are distributed and metabolic products are removed. All these molecules can be monitored by analysis of the extracellular fluid with microdialysis. Monitoring by microdialysis could help guide the therapy of critically ill patients [8].

Microdialysis consists of the continuous collection of extracellular fluid with a microdialysis probe inserted into the tissue and subsequent analysis of this dialysate using a biochemical analyzer. The microdialysis probe, due to its behavior and construction, imitates a blood capillary. The probe can be inserted into any tissue. Numerous studies have described its use in the liver, intestines, muscle and adipose tissue [9-11]. After insertion of the probe into the tissue, the probe comes into contact with the extracellular fluid. The probe is continually flushed with a solution of known composition at a set perfusion speed. This process enables an exchange of substances between the dialysis solution and the extracellular fluid. The dialysate is subsequently collected into microvials and analyzed.

The primary substances that provide information about the degree of anaerobic metabolism and energy use include lactate, pyruvate, glycerol and glucose. The serum lactate level is the result of both lactate production, which occurs during ischemia or stress, and consumption, which occurs as the lactate enters the metabolism in the form of an energetic substrate in tissues such as the liver, heart or brain. The tissue lactate level is solely the result of lactate production. The arterial serum lactate concentration, which is routinely monitored, reflects the condition of the whole organism. However, normal serum lactate values do not provide any information about the regional state of tissues. Patients in shock exhibit a difference in the level of lactate between the tissues and the blood [12]. The most important value is the lactate/pyruvate (L/P) ratio in the extracellular fluid, which serves as a timely marker of emerging ischemia and allows for the monitoring of hemorrhagic shock [13,14]. An increase in the L/P ratio to > 25 indicates the onset of anaerobic metabolism [15]. The L/P ratio may be helpful in discriminating between different mechanisms of hyperlactatemia and to distinguish the anaerobic portion of lactate production. Hyperlactatemia and a simultaneously elevated L/P ratio in patients is associated with a higher mortality than hyperlactatemia with a normal L/P ratio. The L/P ratio is therefore a more complex and precise marker of ischemic conditions [16]. When using the L/P ratio, differences in the lactate levels between ischemic and non-ischemic tissues can be distinguished [17].

Monitoring of the tissue metabolism and microcirculation is a topic in many studies. The main development of this method has been in neurocritical care, and microdialysis has become an important component of multimodal monitoring [18]. The Brain Trauma Foundation guidelines recommend tissue metabolism monitoring. Microdialysis has been shown to be effective in septic shock [19], but septic shock exhibits a different type of microcirculatory dysfunction compared with hemorrhagic shock [20]. Blood transfusion results in a decrease in the L/P ratio in septic patients [21]. The L/P ratio may detect organ ischemia earlier than an increase in intra-abdominal pressure and much earlier than signs of organ failure due to abdominal compartment syndrome [22]. Microdialysis also allows for monitoring of the levels of pharmaceuticals in tissues, the availability of antibiotics and chemotherapeutics, and the immunological state of patients by analyzing tissue cytokines [23-27]. Microdialysis is a sufficiently sensitive method for monitoring anaerobic metabolism and it positively correlates with pro-inflammatory markers [28]. This technique can also be used during surgery or transplantation [29]. A slight modification of the method makes it suitable for monitoring the degree of blood perfusion in individual tissues through blood-flow markers, such as ethanol [30] or urea [31-33]. Microdialysis shows great theoretical potential for use in experimental and clinical medicine.

The primary goal of this study was to determine the correlation between a marker of tissue hypoxia (L/P ratio) and selected parameters of systemic oxygen delivery (Hb; hemoglobin) and oxygen extraction (ScvO_2_; central venous oxygen saturation). The secondary goal was to investigate the severity of tissue hypoxia during the course of care (pre-hospital care, care in the emergency department and operating room care).

## Methods

Polytrauma patients between 18 and 60 years of age were enrolled in this prospective, observational study. All the participants presented with signs of serious traumatic hemorrhagic shock with an estimated blood loss exceeding 1 l. Monitoring was initiated as soon as possible after admission to the emergency department and always within 6 hours. The recorded parameters included demographic data, timeline of care, hemoglobin level, ScvO_2_ level and serum lactate level. A CMA 60 microdialysis probe (CMA Microdialysis AB, Stockholm, Sweden) was placed into the deltoid muscle. We used CMA Perfusion Fluid T1 dialysis solution (a lactate-free Ringer solution). Perfusion was accomplished with a CMA 106 pump at a constant speed of 0.3 μl/min. Subsequent analysis was performed with the CMA Iscus Flex (CMA Microdialysis AB) analyzer using a set of reagents for the analysis of lactate, pyruvate, glycerol and glucose (CMA Reagent Set A). The tissue values were analyzed in 1-hour intervals. Complete blood count and ScvO_2_ were measured in 8-hour intervals, but at least every 2 hours following the administration of blood products, using a biochemical analyzer (Roche Cobas b221 OMNI S). The normal tissue concentrations of the examined metabolites are presented in Table 1. The ethics committee University hospital Ostrava in Czech Republic approved the study and each of the study subjects signed an informed consent form approved by the ethics committee.

**Table 1:** Cut-off values for the tissue concentrations of metabolites in muscle tissue

		
Glucose	5	mmol/l
Lactate	2	mmol/l
Pyruvate	120	μmol/l
Lactate/Pyruvate	>25	
Glycerol	200	μmol/l

(Valid for flow at 0.3 μl/min and a 3-cm membrane length).

We used R software version 2.15.2 for the statistical analysis. The data were divided into time intervals by linear interpolation. A total of 30 time shifts of one hour were assessed independently for hemoglobin and ScvO_2_, resulting in 30^2^ different models. For each model, we tested the dependence with a t-test adjusted by the Holm scheme for multiple comparisons. The results of the tests are depicted in the form of a heat map. On this map, every point represents an individual model for a time shift of the trends in hemoglobin and ScvO_2_ towards the L/P ratio. The colors represent the value of statistical significance, with the deep red color representing statistical significance of p < 0.05. We also investigated whether a correlation existed between the duration of pre-hospital care, the care provided in the emergency department, the duration of surgical procedures, and the L/P ratio (T_ICU_ = sum of prehospital care, care in the emergency department and operative time, it is time before ICU admission).

## Results

A total of 36 patients were included in the statistical analysis.

**Figure 1:**
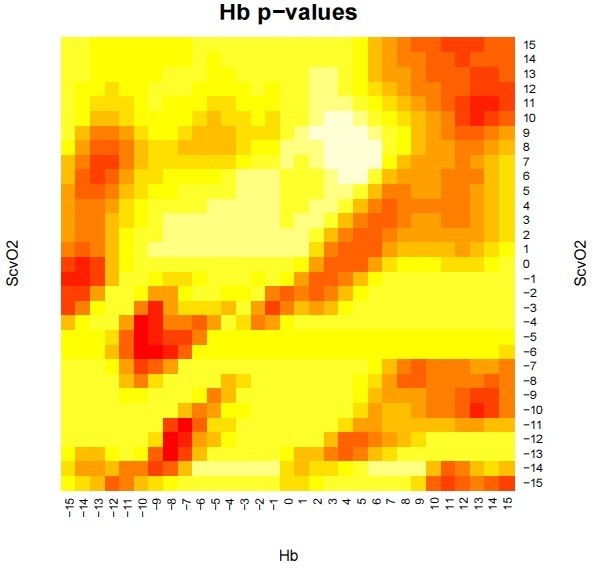
**Correlation of the tissue lactate/pyruvate ratio with time shifts in hemoglobin levels (Hb) and central venous oxygen saturation (ScvO**_**2**_**).** Significance is indicated with colors: the areas of deep red color represent p < 0.05.

The correlation between the L/P ratio and its dependence on the level of hemoglobin and ScvO_2_ in time shifts from -15 to +15 hours is shown in Figure 1. The red areas indicate the strongest correlation (*p* = 0.05). A strong correlation was found between the trend of the L/P ratio and hemoglobin from -7 to -10 hours and in the +13 hour. In other words, the tissue L/P ratio may react to changes in systemic hemoglobin values with a 7- to 10-hour delay. However, it may also manifest up to 13 hours in advance. Similar results were obtained for the ScvO_2_ trend, which showed a strong correlation in hour -10 and between hours +10 and +11.

Table 2 illustrates the times of care; the highest L/P ratios and the lowest hemoglobin values in the first 12 and 24 hours; the time during which the patients had L/P ratios > 25, 30 and 35; and the median and mean L/P ratios in individual patients.

**Table 2:** Monitored values in individual patients

**ID**	**Gender**	**Age**	**ISS**	**PHC**	**E**	**OT**	**Total**	**ICU**	**M**	**L/P max.12 h**	**Hb min.12 h**	**Ls 12 h**	**L/P max.24 h**	**Hb min.24 h**	**Ls 24 h**	**L/P 25**	**L/P 30**	**L/P 35**	**Median L/P**
1MP	M	60	50	70	90	-	160	9	0	12.4	91.7	7.7	17.8	91.2	7.7	3	0	0	20.7
10LJ	M	54	25	30	100	200	330	7	0	32.9	93	4.4	32.9	93	5.2	2.5	0.5	0	16.4
11GM	M	30	41	45	90	250	385	5	0	-	70	7.9	-	70	9.6	24.5	23.8	15	34.9
12HM	M	30	41	55	160	145	360	4	0	23.7	103	3.4	23.7	103	3.4	0	0	0	13.6
13KP	M	33	41	85	115	130	330	15	0	29.8	67	3.2	29.8	67	3.2	7.5	0	0	20.5
14KM	M	51	50	70	90	160	320	28	0	50	63	5	28.5	63	6.2	54.8	18.3	10	24.4
15SM	M	21	50	45	210	100	355	24	0	17.1	72	2.3	17.4	72	2.3	12.5	2.7	1.3	18.2
16KB	M	55	25	60	60	70	190	1	1	368	9	17	368	9	17	0	0	0	368.9
17HM	M	20	29	50	90	210	350	17	0	33.9	83	4.3	33.9	75	4.3	0.8	0.3	0	17.9
18SD	M	30	59	65	80	105	250	53	0	63.7	47	13.4	63.7	47	13.4	21.3	11	3	19,1
19BZ	M	19	25	50	25	100	175	3	0	22.9	51	8.1	22.9	51	8.1	0.3	0	0	15.6
2KS	F	31	41	105	155	0	260	25	0	26	74	2.7	26	74	2,7	51.5	21.5	0.3	26.3
20BR	M	22	57	80	70	240	390	62	0	30.9	66	10.6	30.9	66	10.6	28	17.8	2.3	24.8
21NM	F	53	66	30	160	110	300	77	0	54	67	3	54	67	3.2	22.8	3.8	2.5	21
22JS	M	38	66	45	140	-	185	21	0	65	61	16	65	61	16	71	66	43.5	34.1
23SM	M	22	50	45	205	210	460	23	0	19	51	2.8	19	51	2.8	0	0	0	13.7
24PP	M	40	20	50	70	100	220	10	0	38.1	77	3.1	38.7	58	4.9	10.5	9.8	6.8	16.7
25SJ	M	42	45	60	110	-	170	7	0	19.2	126	5.2	24.2	123	5.2	0	0	0	20.5
26SF	M	62	57	55	105	480	640	7	0	12.7	71	4.6	12.7	64	6.2	5	0	0	16.8
28VJ	M	38	50	50	70	160	280	3	0	38.6	99	3.9	38.6	99	3.9	4	3	0.5	14.1
29ZM	M	23	34	40	145	-	185	29	0	-	-	-	20.3	66	6.9	16.5	9.5	5.3	16.2
3FR	M	39	41	-	-	200	200	10	0	49.9	76	2.5	52.9	76	3.9	75	64.2	53.5	38.4
30AV	M	55	59	30	130	180	340	21	1	43.8	82	9.5	43.8	82	11.8	67.8	49.2	24.8	32
32BJ	M	30	57	90	130	160	380	23	0	4	90	2.7	10	90	2.7	2	0	0	15.5
33HP	M	40	43	95	60	60	215	12	0	-	91	7.6	-	91	7.6	1	0	0	18.6
34PJ	M	50	27	40	160	370	570	22	0	28.5	69	7.3	28.5	69	7.3	6	2.3	1	19.8
35DM	M	23	48	115	65	165	345	26	0	28	79	1.4	28	79	1.4	9	0	0	16.6
36FP	M	67	50	80	155	-	235	7	1	16.7	79	6.3	16.7	79	8.8	0	0	0	13.8
38LM	F	73	50	75	60	150	285	16	0	-	87	5	24.1	87	5	2.5	0	0	21.8
39GP	F	32	41	50	40	90	180	28	0	19	70	7.4	19	70	7.4	0	0	0	12.5
40OK	M	49	41	55	80	160	295	4	0	15.9	87	4.6	15.9	87	4.6	0	0	0	10.9
41BV	M	64	57	70	80	140	290	2	1	99	48	12	99	48	12	1.8	1	1	19.1
5BB	M	34	48	40	80	170	290	6	0	-	73	5.5	19.9	73	5.5	49.5	22	4.2	29
6FL	F	27	34	120	100	260	480	7	0	20.7	60	6.2	27.5	60	6.2	5	0	0	16.6
8PL	M	43	34	160	135	200	495	9	0	53.2	81	1	53.2	64	3	28.2	14.5	7.5	24.5
9KR	M	32	29	135	105	120	360	14	0	44.4	105	1.6	44.4	97	1.6	47.5	45.5	39.5	37

ISS, International Severity Score; PHC, prehospital care time; E, emergency care time; OT, operation time; Total, overall care time before ICU care; ICU, overall time (days) in ICU; M, 30-day mortality (0 = no, 1 = yes); L/P max.12 h, maximal value of L/P ratio in the first 12 hours; Hb min.12 h, minimal value of hemoglobin in the first 12 hours (g/l); Ls 12H, maximal value of serum lactate in the first 12 hours (mmol/l) (similarly for the first 24 hours); L/P 25, number of hours during which the value of the L/P was >25 (similarly, the L/P 30 and L/P 35 indicate >30 and >35, respectively).

**Figure 2:**
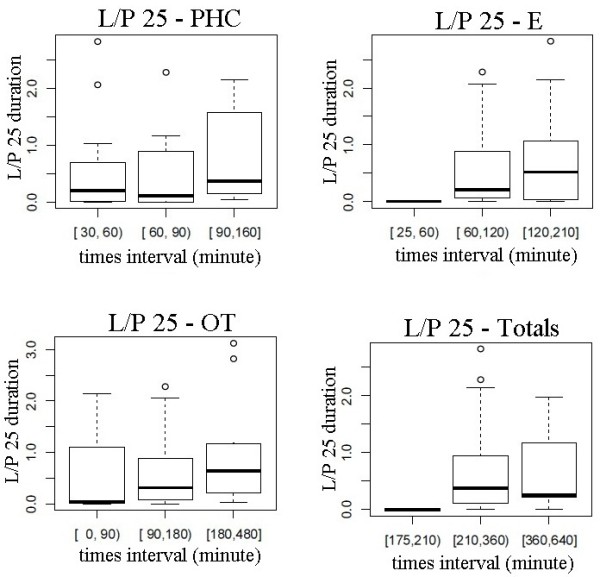
**Duration of tissue ischemia according to the duration of the treatment period.** PHC, prehospital care; E, emergency care; OT, operation time; Totals, summary of PHC + E + OT. Duration of care in intervals on the X-axis. Duration of L/P >25 on the Y-axis (relative units).

Figure 2 presents the duration of tissue ischemia with L/P ratios exceeding 25 according to the length of care. In the T_ICU_ ≤ 240 minutes treatment group, the median value of the maximal L/P ratio in the first 12 resp. 24 hours was 22.9 resp. 23.55. For the group T_ICU_ > 240 minutes, the corresponding values of the maximal L/P ratio was 30.3 in the first 12 hours and 29.15 in the first 24 hours. The duration of the period in which the L/P ratio was > 25 was 1 hour in the faster treatment group and 7.5 hours in the group with the longer treatment duration (*p* = 0.05). The severity of the trauma was comparable in both groups (the ISS score in the T_ICU_ ≤ 240 minutes group was 41, and it was 45 in the T_ICU_ > 240 minutes group), and both groups had the same average age (41 years in the T_ICU_ ≤ 240 minutes group and 39 years in the T_ICU_ > 240 minutes group).

## Discussion

If we attempt to draw clinical conclusions from the monitored tissue values, it is very important to understand that the tissue values represent the state of cells in the monitored area only. The aim of this study was determine how these values correspond to the global parameters that are routinely measured in the intensive care unit, particularly hemoglobin levels and ScvO_2_. The microcirculation of the peripheral muscle tissue is affected very early in patients with serious forms of traumatic hemorrhagic shock [34]. The damage is very intense and requires a long period of recovery [4]. Tissue values may be influenced by the centralization of the circulation or by a stress-induced (i.e., non-ischemic) increase in lactate [35]. The interpretation of lactate values [12] and the L/P ratio [21] may also be guided by using the studies of patients with septic shock. In those studies, the highest tissue lactate values preceded the highest serum lactate values by 4 hours and the L/P ratio decreased in reaction to transfusion.

The most important parameter is the interpretation of the lactate values, particularly the L/P ratio, which is a generally accepted marker of ischemia [36]. Both lactate and pyruvate are influenced in the same way by local factors, such as the centralization of circulation that causes hypoperfusion of the peripheral tissue. The interpretation of this ratio is simpler than explaining the trend of isolated tissue lactate. The most frequently presented threshold value for the L/P ratio in ischemia is 25. Numerous papers in the literature have taken into consideration a higher trigger point of approximately 35 or 40 [37]. It seems that the best strategy for overtly ischemic conditions is to use L/P values over 30.

Figure 1 demonstrates that the L/P ratio trend may react to increase in systemic hemoglobin levels with a delay of 7 to 10 hours. Hemoglobin is one of the main determinants of oxygen delivery, and low values in conjunction with hypovolemia may cause tissue hypoxia. One of the first compensatory responses is centralization of the circulation, followed by increased cardiac output (especially via tachycardia) and increased oxygen extraction (via decreased ScvO_2_). When the oxygen demand is higher than the oxygen extraction limit, an oxygen debt and further tissue ischemia will develop [38]. Normalization of the increased L/P ratio by transfusion occurred after a delay of the aforementioned 7 to 10 hours, which indicated restoration of aerobic metabolism in the tissues and start of redemption of the oxygen debt. Similar changes were observed in increased ScvO_2_ levels with an L/P ratio delay of up to 10 hours. Ischemia of the peripheral muscle tissue was eliminated by a significant time interval after transfusion.

Increase in the L/P ratio may precede the ScvO_2_ decrease by 10 or 11 hours. Exceeding the limit of oxygen extraction in tissues was reflected by the increased L/P ratio. We were able to detect this condition in advance of the ScvO_2_, which is one of main indicators for transfusion [39].

We also observed changes in the L/P trend preceding a change in the hemoglobin level up to 13 hours in advance. Shock and centralization of the circulation could cause high and further increasing L/P ratios due to tissue ischemia. Fluid administration, which is routinely used as a first-line treatment of hypovolemic shock, can cause hemodilution and decreased hemoglobin. The development of anemia is preceded by an increase in the L/P ratio. An increase in the L/P ratio is an early warning sign of inadequate oxygenation in tissues and should lead to early control of the hemoglobin level, ScvO_2_ and other factors of oxygen delivery such as hemodynamic parameters.

Table 2 and Figure 2 show the dependence of the length of care on the L/P ratio within the first 12 and 24 hours. The timeline of care is divided into individual stages, i.e., pre-hospital care, care provided in the emergency department, and surgical time. Patients who are treated within a shorter time frame (T_ICU_ ≤ 240 minutes) have lower maximal L/P ratios and generally have a shorter duration of tissue ischemia.

## Conclusion

Microdialysis is a modern method that is on the border of experimental and clinical practice and the values monitored with this technique provide new insights into the condition of cells in critically ill patients. We can observe the dependence of the L/P ratio on the hemoglobin level and ScvO_2_, which in turn inform us about anaerobic tissue metabolism and allow us to alter treatment. The L/P ratio was useful to identify tissue ischemia and could estimate the effectiveness of fluid resuscitation. Normalization of an elevated L/P ratio could help guide resuscitation efforts and could be an indicator for fluid administration. An increased L/P ratio is an early warning sign of inadequate tissue oxygenation and should lead to more detailed hemodynamic and laboratory monitoring. This information cannot usually be obtained from global markers. Microdialysis has immense potential and unlimited possibilities for monitoring the various molecules in many organs and tissues. The search for the optimal marker of adequate resuscitation continues.

## Abbreviations

ScvO2: Central venous oxygen saturation; L/P: Lactate/pyruvate ratio; SOFA: Sequential organ failure assessment score; ICU: Intensive care unit; DO2: Oxygen delivery; VO2: Oxygen consumption.

## Competing interests

The authors declare that there are no other competing interests.

## Authors’ contribution

FB collection and analysis of data, interpretation and publication of data, co-investigator. LP principal investigator, publication of results. All authors read and approved the final manuscript.
